# Association Between HIV Infection Duration and Left Ventricular Concentric Remodeling and Myocardial Fibrosis: A Cross‐Sectional Study Using Cardiac MRI

**DOI:** 10.1002/jmri.70395

**Published:** 2026-06-11

**Authors:** Chunyan Deng, Wei Sun, Tianhu Gao, Haixia Chen, Lu Liu, Ran Li

**Affiliations:** ^1^ Department of Radiology, Banan Hospital Chongqing Medical University Chongqing China; ^2^ Department of Ultrasound, Banan Hospital Chongqing Medical University Chongqing China; ^3^ Department of Infectious Diseases, Banan Hospital Chongqing Medical University Chongqing China

**Keywords:** cardiac, concentric remodeling, extracellular matrix volume index, HIV, myocardial fibrosis

## Abstract

**Background:**

Antiretroviral therapy has transformed HIV into a chronic condition, yet cardiac injury remains a major issue.

**Purpose:**

To assess the ability of cardiac MR imaging markers to predict cardiac injury and disease progression in HIV‐infected individuals.

**Study Type:**

Prospective.

**Subjects:**

Eighty‐three HIV‐infected participants (short‐duration infection < 5 years, *n* = 43; long‐duration ≥ 5 years, *n* = 40) and 34 matched HIV‐negative controls.

**Field Strength/Sequence:**

1.5 T; SSFP cine and MOLLI sequences.

**Assessment:**

Left ventricular (LV) end diastolic volume indexed to body surface area (LVEDVi), ejection fraction (LVEF), mass (LVM), myocardial mass‐to‐volume ratio (MVR), and global strains were determined from SSFP cine data. Extracellular volume (ECV) and myocardial extracellular matrix volume index (ECMVi) were determined from pre‐ and post‐contrast myocardial T1 values.

**Statistical Tests:**

ANOVA, Kruskal–Wallis, post hoc tests, correlation, and multivariable logistic regression; *p* < 0.05 indicated statistical significance.

**Results:**

Both the HIV groups showed significantly elevated ECV (short: median 28.02 [25.40–31.86]%; long: median 28.18 [24.40–37.99]% vs. 25.07% ± 5.61%) and myocardial native T1 (1863.35 ± 300.29 ms; median 1963.00 [1807.00, 2072.25] ms vs. median 1734 [1309.75–1981.50] ms) compared to controls. The long‐duration group exhibited significantly higher ECMVi (14.73 ± 4.59 mL/m^2^ vs. controls: 10.52 ± 2.82 mL/m^2^ and short: 12.32 ± 2.83 mL/m^2^) and MVR (0.78 ± 0.16 vs. 0.61 ± 0.11 g/mL and median 0.63 [0.55–0.69] g/mL), significantly reduced LVEDVi (65.44 ± 13.21 vs. 73.42 ± 10.99 and 72.08 ± 10.62 mL/m^2^) and significantly diminished global radial strain (29.82% ± 6.28% vs. controls: 36.18% ± 6.39% and short: median 33.16 [28.44–37.52]%).HIV duration correlated positively with ECMVi and MVR (*r* = 0.277 and 0.474, respectively), and significantly negatively correlated with global radial strain (*r* = −0.244). Two robust MRI models were validated: ECV/native T1/fasting blood glucose (FBG) for early HIV (AUC = 0.852) and LVEDVi/ECMVi/FBG for duration stratification (AUC = 0.844), with stable performance through bootstrapping and calibration.

**Conclusion:**

ECV, myocardial native T1, and FBG can predict short‐duration HIV infection, while a multimodal model (LVEDVi, ECMVi, and FBG)stratifies duration. However, prospective validation is necessary.

**Evidence Level:**

2.

**Technical Efficacy:**

Stage 3.

## Plain Language Summary

HIV patients face hidden heart damage even without symptoms. We used cardiac MRI to detect early injury in 83 HIV‐infected individuals (short‐ and long‐duration infections) and 34 healthy controls. The results showed that early‐stage HIV causes subtle inflammation (higher ECV and native T1), while longer infection leads to heart stiffening (higher ECMVi, smaller chamber size, and reduced radial strain). A model combining ECV, native T1, and blood sugar accurately identified early infection. Another model using LVEDVi, ECMVi, and blood sugar helped distinguish infection duration. These MRI markers may enable earlier intervention to prevent heart failure in HIV patients.

## 1 Introduction

Antiretroviral therapy (ART) has resulted in human immunodeficiency virus (HIV) infection being a manageable chronic condition, with markedly improved life expectancy for affected individuals [1]. However, complications associated with long‐term immune activation, chronic inflammation, and treatment, particularly cardiovascular disease, have emerged as leading causes of morbidity and mortality in this population [1]. Despite effective viral load control with ART, people living with HIV (PLWH), even asymptomatic individuals, exhibit extensive subclinical myocardial pathologies, such as myocardial fibrosis, inflammatory remodeling, and metabolic disturbances [2, 3]. These changes, which are considered important pathological bases for heart failure, arrhythmias, and sudden death [4, 5, 6], often precede decreases in left ventricular ejection fraction (LVEF), necessitating more sensitive detection methods for early warning.

Multiple cardiac imaging studies have shown complex features of structural heart disease, including impaired systolic and diastolic function, reduced myocardial strain, and increased left ventricular (LV) mass with remodeling [3, 6, 7, 8]. These findings provide insights into the underlying pathophysiological mechanisms. Additionally, cardiac MR imaging results have shown significantly increased incidence and extent of microvascular disease, cardiac steatosis, and myocardial scar formation among this population [7, 8].

However, in these previous studies, healthy volunteers were predominantly used as controls, and systematic stratification on the basis of infection duration is lacking. Furthermore, sex bias exists in research populations, with an underrepresentation of women [9], limiting comprehensive mechanistic analysis and assessment of overall heterogeneity [10]. Consequently, it remains unclear how subclinical cardiac injury evolves with the duration of infection in treated, asymptomatic PLWH, and whether sensitive imaging biomarkers associated with infection time can be identified.

Thus the aim of this study was to evaluate differences in myocardial structure, function, and histological characteristics between controls and asymptomatic PLWH with short‐duration (< 5 years) and long‐duration (≥ 5 years) HIV infection. A further aim was to identify sensitive biomarkers significantly associated with infection duration which could potentially provide a basis for early intervention.

## 2 Materials and Methods

### 2.1 Patients

This study was approved by the institutional ethics committee, and written informed consent was obtained from all the participants (BNLL‐KY‐2024‐001). All the procedures adhered to the principles outlined in the Declaration of Helsinki (2013).

The study was a prospective, cross‐sectional, observational, investigator‐led analysis of the independent predictive value of HIV infection duration in HIV‐infected individuals. Eighty three participants with a confirmed diagnosis of HIV infection were enrolled from October 2022 to May 2025, and all patients received standardized ART treatment for ≥ 6 months. They were referred from the specialized HIV Center at Banan Hospital affiliated with Chongqing Medical University in China. This referral was initiated to evaluate potential cardiac involvement, given the elevated risk of cardiovascular disease associated with HIV. Notably, owing to the recognized high incidence of nonischemic cardiac conditions in this population, the exhibition of typical cardiac symptoms was not a prerequisite for inclusion in the study. The exclusion criteria included contraindications to MR imaging, such as the presence of MR‐unsafe implanted devices (*n* = 0), a documented history of allergic reactions to gadolinium‐based contrast agents (*n* = 1), an inability to maintain breath holding during the MRI examination (*n* = 5), and a diagnosis of myocardial disease, which encompassed conditions such as myocardial infarction, cardiomyopathy, or severe arrhythmias, as substantiated by a 12‐lead electrocardiogram or clinical history. Additionally, individuals younger than 18 were excluded. However, an estimated glomerular filtration rate (eGFR) of less than 30 mL/min/1.73 m^2^ was not considered a contraindication, aligning with the prevailing guidelines regarding the diagnostic application of gadolinium‐based contrast agents [11]. The enrolled patients were classified into two groups according to how long they had received ART: a long‐duration HIV group, with an infection duration of 5 years or longer (*n* = 40), and a short‐duration HIV group, with an infection duration of less than 5 years (*n* = 43). Additionally, 34 sex‐ and age‐matched healthy volunteers were included as a control group.

### 2.2 Demographic Characteristics and Laboratory Parameters

The recorded data included age (years), sex, body mass index (BMI, kg/m^2^), and body surface area (BSA), calculated using the DuBois formula [12]. Biochemical parameters, including fasting blood glucose (FBG), low‐density lipoprotein cholesterol (LDL‐C), total cholesterol (TC), and triglyceride levels, were measured. Data on CD4+ T‐cell counts before ART, viral loads before MR examination, medical history‐taking, specific ART regimens used and ART duration (years) were obtained from clinical records. Antiretroviral drugs primarily include nucleoside reverse transcriptase inhibitors (NRTIs), non‐nucleoside reverse transcriptase inhibitors (NNRTIs), and integrase strand transfer inhibitors (INSTIs).

### 2.3 MR Imaging Protocol

All participants underwent MRI in the supine position in a whole‐body 1.5 T (Signa Voyager; GE Healthcare, Milwaukee, USA) Imaging was conducted during end‐expiratory breath holding. MR examinations included assessments of cardiac function, native T1 mapping and postcontrast T1 mapping. Cardiac function was assessed using a steady‐state free‐precession (SSFP). All scans utilized prospective electrocardiographic (ECG) gating. The typical breath‐hold duration per slice was approximately 8–12 s. Acquisition parameters included a repetition time (TR) of 6.56 ms, an echo time (TE) of 3.02 ms, a slice thickness of 8 mm, a field of view (FOV) of 350 × 350 mm^2^, a matrix size of 164 × 288 pixels, a temporal resolution (acquired) of 62 ms, and a flip angle of 65°. Signal averages were set to 1 for breath‐hold acquisitions and increased to 3 for free‐breathing navigator‐gated scans to compensate for motion. Eight to 12 contiguous short‐axis cine datasets were obtained, in addition to 2‐, 3‐, and 4‐chamber datasets extending from the level of the mitral valve annulus to the apex of the LV. Pre‐ and post‐contrast T1 mapping was performed using a modified look–locker inversion recovery (MOLLI) sequence in 3 short‐axis planes: basal, mid‐ventricular, and apical. The imaging parameters were: TR, 3.7 ms; TE, 1.1 ms; flip angle, 35°; matrix acquisition size, 160 × 160; FOV, 350 × 350 mm; slice thickness, 8 mm; TR/TE, 3.55/1.53 ms; trigger delay, 300 ms; and number of views per segment, 24. Signal intensity curves were processed with a curve fitting technique to generate T1 maps. Post‐contrast T1 mapping was performed at the same slice locations approximately 25 min after the contrast injection (Gadolinium–DTPA, Shandong Weizhi Baike Pharmaceutical Co. Ltd., Zaozhuang, China).

### 2.4 Image Analysis

All analyses were conducted using an offline commercial software package (cvi42, version 5.9.1; Circle Cardiovascular Imaging, Calgary, Canada). All data were evaluated by two observers (C.H.X. and G.T.H. with 8 and 12 years of experience, respectively), each with 5 years of experience in the field of cardiac MRI.

### 2.5 Evaluation of Myocardial T1 Values and ECV Quantification

**FIGURE 1:**
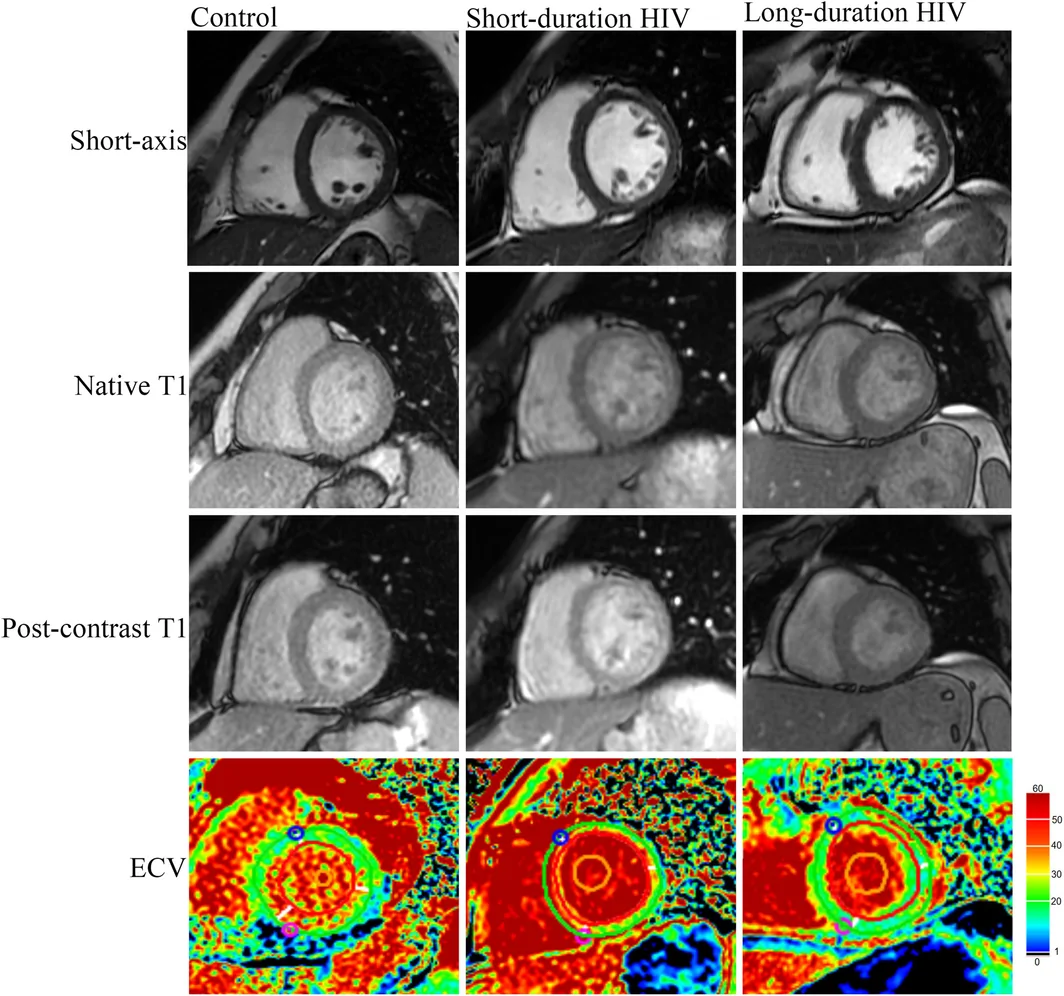
Representative image panels comparing the three groups (Control, short‐duration HIV, and long‐duration HIV) across all four sequences: Short‐axis, native T1, post‐contrast T1, and ECV.

For each short‐axis image (base, mid‐ventricular, apex), Myocardial segmentation followed the AHA 16‐segment model (6 basal, 6 mid‐ventricular, 4 apical, excluding the true apex). ROIs were automatically delineated along endocardial and epicardial borders to minimize partial volume effects, excluding papillary muscles and artifacts. Native T1 values were quantified per segment and per slice, then averaged across all 16 segments to produce a single global myocardial T1 per subject, consistent with population‐based outcome prediction protocol [13]. No segment‐level results are reported, and segmentation was manually defined per the AHA model. The extracellular volume (ECV) was calculated as follows: ECV = (1/T1 myo post − 1/T1 myo pre) × (1 − Hct)/(1/T1 blood post − 1/T1 blood pre). Hematocrit (Hct) measurements were obtained from venous blood samples collected concurrently with magnetic resonance (MR) imaging, and the measured venous hematocrit (vHct) values were used in the standard ECV calculation. Samples were processed using automated hematology analyzers, with results reported as percentages and converted to decimals (e.g., 42% → 0.42) for ECV calculation. The myocardial extracellular matrix volume index (ECMVi) was calculated as the ECV multiplied by the left ventricular mass (LVM) divided by the BSA (ECMVi = ECV × LVM/BSA), while the myocardial cell volume index (CVi) was calculated by subtracting the ECV from 1 and multiplying the result by the LVM divided by the BSA (CVi = [1 − ECV] × LVM/BSA) (Figure 1).

### 2.6 MR Data Analysis and Definitions

To assess LV functional parameters, the endocardial and epicardial borders were delineated in consecutive short‐axis slices during both the end‐diastolic and end‐systolic phases. Afterwards, the left ventricular end‐diastolic volume (LVEDV), left ventricular end‐systolic volume (LVESV), stroke volume (SV), left ventricular ejection fraction (LVEF), cardiac output (CO) and left ventricular mass (LVM) were calculated automatically. The LVEDV, SV, LVESV, CO and LVM values were adjusted by the BSA to obtain the left ventricular end‐diastolic volume index (LVEDVi), stroke volume index (SVi), left ventricular end‐systolic volume index (LVESVi), cardiac index (CI)and myocardial mass index (MMi), respectively. Additionally, the left ventricular remodeling index was derived by dividing the LVM by LVEDVi.

In the two‐chamber and four‐chamber cine datasets, the left atrial (LA) contour was automatically delineated to measure the left atrial maximum volume (LAVmax), left atrial pre‐atrial contraction volume (LAVpre‐A), and left atrial minimum volume (LAVmin). The following parameters were subsequently calculated: total left atrial ejection fraction (LATEF) = (LAVmax − LAVmin) × 100%/LAVmax; passive left atrial ejection fraction (LAPEF) = (LAVmax − LAVpre‐A) × 100%/LAVmax; and active left atrial ejection fraction (LAAEF) = (LAVpre‐A − LAVmin) × 100%/LAVpre‐A.

LV and LA myocardium strain parameters were determined from the SSFP cine datasets. During the end‐diastolic phase, which served as the reference, the endocardial and epicardial contours were delineated in long‐axis 2‐chamber, 4‐chamber, and sequential short‐axis slices, employing a three‐dimensional (3D) tissue tracking module. Next, the LV global radial, circumferential and longitudinal peak strains (LV‐GRS, LV‐GCS and LV‐GLS, respectively), the LV global radial, circumferential and longitudinal peak systolic strain rates (LV SRs‐R, LV SRs‐C and LV SRs‐L, respectively), the LV global radial, circumferential and longitudinal peak early diastolic strain rates (LV SRe‐R, LV SRe‐C and LV SRe‐L, respectively), as well as the left atrial peak global longitudinal strain (LA PGLSt), were automatically derived.

### 2.7 Reproducibility

To assess the reliability of the measurement results, MR datasets from 20 randomly selected subjects were independently analyzed by two radiologists (L.R. and D.C.Y., with 18 and 14 years of experience, respectively), with an interval of at least 1 week between the evaluations. One radiologist (D.C.Y.) analyzed the same datasets again 1 month later to evaluate the intraobserver agreement.

### 2.8 Statistical Analysis

Statistical analyses were performed using SPSS 21.0 (IBM Corp.) and R 4.3.1. Categorical variables are presented as frequencies (percentages), and between‐group differences were assessed using the *χ* ^2^ test. The normality of continuous variables was evaluated using the Kolmogorov–Smirnov test. Normally distributed data are expressed as the mean ± standard deviation. Group comparisons were performed using one‐way ANOVA (assuming homogeneity of variance) with Bonferroni‐adjusted post hoc tests. Correlations were analyzed using Pearson's coefficient (*r*). Nonnormally distributed data are expressed as medians (interquartile ranges) [M (Q1, Q3)]. Group comparisons were performed using the Kruskal–Wallis *H* test with Bonferroni‐adjusted Dunn's post hoc test. Correlations were analyzed using Spearman's coefficient (*r*).

To construct the prediction model, univariate analysis was first performed on the candidate variables (screening criterion: (*p* < 0.05)). Statistically significant variables were then entered into a multivariate logistic regression analysis to identify independent predictors and establish the final prediction model. The discriminative performance was evaluated using the receiver operating characteristic (ROC) curve, with the area under the curve (AUC) calculated. The optimal cutoff value was determined based on the Youden index (i.e., the maximum value of sensitivity + specificity − 1), and the corresponding sensitivity, specificity, positive predictive value (PPV), and negative predictive value (NPV) were reported. To assess the model's stability, internal validation was performed using the bootstrap method (1000 resamples). A calibration curve along with a decision curve analysis (DCA), was plotted to comprehensively evaluate the model's calibration and clinical applicability.

Intraclass correlation coefficients (ICCs) with 95% confidence intervals (CIs) were used to assess intra‐ and inter‐observer reliability for continuous measurements. Based on the ICC estimate's 95% confidence interval, reliability was classified as poor (< 0.5), moderate (0.5–0.75), good (0.75–0.90), and excellent (> 0.90) [14]. Bland–Altman analysis was performed to quantify mean bias and 95% limits of agreement between repeated measurements; representative quantitative parameters (e.g., ECMVi, MVR, and LV‐GRS) were selected to generate scatter plots visualizing measurement agreement. All tests were two‐tailed, and *p* < 0.05 was considered to indicate statistical significance.

## 3 Results

### 3.1 Baseline Characteristics

Compared with the healthy control group, the HIV‐infected groups had significantly higher TC and LDL‐C levels. Significant differences in FBG levels were observed among the three groups: the control group presented the lowest levels, the long‐duration HIV group presented intermediate levels, and the short‐duration HIV group presented the highest levels. The HIV short‐duration group had a median of 3.00 years, while the long‐duration group had 8.00 years. Most patients received ART based on two NRTIs plus a third agent. The long‐duration group had more patients on a 2 NRTI + NNRTI regimen (87.50% vs. 72.09%), with fewer on a 2 NRTI + INSTI regimen (7.5% vs. 14.0%) and other regimens (5.0% vs. 14.0%). No significant differences in baseline demographic characteristics, including age, sex or BMI were observed among the three groups (*p* = 0.128, *p* = 0.119, and *p* = 0.467, respectively). However, BSA was significantly lower in the control group than in both the short‐duration HIV group and the long‐duration HIV group (Table 1).

**TABLE 1:** Demographics and clinical characteristics of the control and HIV groups.

Variables	Control group (*N* = 34)	Short‐duration HIV group (*N* = 43)	Long‐duration HIV group (*N* = 40)	*p*	Post hoc pairwise comparison *p*
Age (years)	42.88 ± 12.75	46.98 ± 11.29	48.15 ± 10.52	0.128^a^	—
Male [*n* (%)]	13 (38.2%)	26 (60.5%)	23 (57.5%)	0.119^c^	—
BMI (kg/m^2^)	24.79 ± 2.03	25.55 ± 3.28	25.34 ± 2.59	0.467^a^	—
BSA (m^2^)	1.65 ± 0.10	1.73 ± 0.12	1.67 ± 0.10	**< 0.001** ^a^	0 vs. 1: 0.002; 0 vs. 2: < 0.001
Hypertension [*n* (%)]	2 (5.9%)	3 (7.0%)	4 (10.0%)	0.784^c^	
Smoking [*n* (%)]	10 (29.4%)	17 (39.5%)	16 (40.0%)	0.566^c^	
Syphilis [*n* (%)]		9 (20.9%)	11 (27.5%)	0.484^c^	
Initial CD4 count (cells/μL)	—	207 (127, 383)	244 (170, 338)	0.439^b^	—
CD4 count before MR examination (cells/μL)	—	480 (330, 646)	510 (399, 628)	0.224^b^	—
ART duration (years)	—	3.00 (1.00, 4.00)	8.00 (6.25, 10.00)	< 0.001^b^	
ART regimen [*n* (%)]	—	—	—	0.207^c^	—
2 NRTI + 1 NNRTI	—	31 (72.09%)	35 (87.50%)	—	—
2 NRTI + 1 INSTI	—	6 (13.95%)	3 (7.50%)	—	—
Others	—	6 (13.95%)	2 (5.00%)		
FBG (mmol/L)	4.85 (4.60, 5.23)	5.40 (5.20, 6.00)	5.30 (4.83, 5.50)	**< 0.001** ^b^	0 vs. 1: < 0.001; 0 vs. 2: 0.008; 1 vs. 2: 0.032
Triglycerides (mmol/L)	1.21 (0.92, 1.76)	1.44 (1.20, 2.51)	1.50 (1.15, 2.31)	0.102^b^	—
TC (mmol/L)	3.92 (3.39, 4.45)	4.74 ± 0.90	4.66 ± 0.75	**< 0.001** ^b^	0 vs. 1: < 0.001; 0 vs. 2: < 0.001
LDL‐C (mmol/L)	2.43 ± 0.63	2.97 (2.62, 3.37)	2.91 ± 0.81	**0.006** ^b^	0 vs. 1: 0.003; 0 vs. 2: 0.011

*Note:* Statistical methods: Continuous variables are presented as mean ± SD for normally distributed data or median (Q1, Q3) for non‐normally distributed data. Post hoc analysis: Bonferroni correction applied for pairwise comparisons. *p* < 0.05 was considered to indicate statistical significance, Bold values denote statistically significant differences (*p* < 0.05). Groups: 0 = control; 1 = short‐duration HIV group; 2 = long‐duration HIV group.

Abbreviations: ART, Antiretroviral Therapy; BMI, body mass index; BSA, body surface area; FBG, fasting blood glucose; INSTI, integrase strand transfer inhibitor; LDL‐C, low‐density lipoprotein cholesterol; MR, magnetic resonance; NNRTI, nonnucleoside reverse transcriptase inhibitor; NRTI, nucleoside reverse transcriptase inhibitor; TC, total cholesterol.

^a^ One‐way ANOVA, mean ± SD.

^b^ Kruskal–Wallis *H* test, median (Q1, Q3).

^c^ Chi‐square test (*χ* ^2^), *n* (%).

Comparative analysis of HIV‐specific clinical parameters showed no significant differences between the short‐ and long‐duration HIV groups, including baseline CD4^+^ T‐cell counts and CD4^+^ T‐cell counts, prior to the current MR examination (*p* = 0.224 and *p* = 0.439, respectively) (Table 1).

### 3.2 Myocardial Tissue Characteristics

Myocardial native T1 values were significantly greater in both the long‐ and short‐duration HIV groups than in the control group (median 1963 ms [IQR: 1807, 2072.25] and 1863.35 ± 300.29 ms, respectively, vs. median 1734 ms [IQR: 1309.75, 1981.5]). No significant difference in T1 values was found between the long‐ and short‐duration HIV groups (*p* = 0.292). Similarly, the ECV was significantly greater in the long‐ and short‐duration HIV groups than in the control group (median 28.18% [IQR: 24.40, 37.99] and median 28.02% [IQR: 25.40, 31.86], respectively, vs. 25.07% ± 5.61%), with no significant difference between the long‐ and short‐duration HIV groups (*p* = 0.640). Additionally, the ECMVi in the long‐duration HIV group (14.73 ± 4.59 mL/m^2^) was significantly greater than that in the control group (10.52 ± 2.82 mL/m^2^) and that in the short‐duration HIV group (12.32 ± 2.83 mL/m^2^). However, the CVi did not significantly differ among the groups (*p* = 0.575) (Table 2).

**TABLE 2:** Comparison of MR data between the control and HIV groups.

Variables	Control group (*N* = 34)	Short‐duration HIV group (*N* = 43)	Long‐duration HIV group (*N* = 40)	*p*	Post hoc pairwise comparison *p*
Myocardial native T1 (ms)	1734 (1309.75, 1981.50)	1863.35 ± 300.29	1963.00 (1807.00, 2072.25)	**0.012** ^b^	0 vs. 1: 0.049; 0 vs. 2: 0.003
Blood native T1 (ms)	3207 (2176.50, 3587.50)	3312.81 ± 523.01	3495.33 ± 625.65	**0.006** ^b^	0 vs. 2: 0.001
Myocardial post‐contrast T1 (ms)	635.18 ± 209.98	542.58 ± 146.68	569.00 (450.00, 716.75)	0.081^b^	—
ECV (%)	25.07 ± 5.61	28.02 (25.40, 31.86)	28.18 (24.40, 37.99)	**0.005** ^b^	0 vs. 1: 0.008; 0 vs. 2: 0.002
ECMVi (mL/m^2^)	10.52 ± 2.82	12.32 ± 2.83	14.73 ± 4.59	**< 0.001** ^a^	0 vs. 2: < 0.001; 1 vs. 2: 0.007
CVi (mL/m^2^)	31.69 ± 7.21	31.04 ± 8.11	32.20 (25.33, 41.04)	0.575^b^	—
MVR (g/mL)	0.61 ± 0.11	0.63 (0.55, 0.69)	0.78 ± 0.16	**< 0.001** ^b^	0 vs. 2: < 0.001; 1 vs. 2: < 0.001
LV EDVi (mL/m^2^)	73.42 ± 10.99	72.08 ± 10.62	65.44 ± 13.21	**0.007** ^a^	0 vs. 2: 0.012; 1 vs. 2: 0.033
LV ESVi (mL/m^2^)	27.54 ± 6.15	28.02 ± 6.56	26.12 ± 6.74	0.394^a^	—
SVi (mL/m^2^)	43.54 ± 7.60	43.60 ± 6.96	40.84 ± 8.86	0.206^a^	—
LV EF (%)	61.24 ± 4.13	61.05 ± 5.58	61.03 ± 4.97	0.981^a^	—
LV CI (L/min/m^2^)	2.97 ± 0.54	3.18 ± 0.63	2.83 ± 0.72	0.052^a^	—
LVM (g)	73.29 ± 17.06	79.4 ± 20.12	90.13 ± 24.06	0.003^a^	0 vs. 2: 0.002
MMi (g/m^2^)	44.32 ± 9.00	45.53 ± 10.09	50.03 (42.87, 57.01)	0.064^b^	—
LAVmin (mL)	18.25 ± 6.68	23.44 ± 6.63	23.79 ± 6.87	**0.001** ^a^	0 vs. 1: 0.003; 0 vs. 2: 0.002
LAVmax (mL)	53.46 ± 16.07	55.39 ± 13.15	55.52 ± 14.57	0.797^a^	—
LAVpre‐A (mL)	31.12 ± 9.52	34.30 ± 8.47	34.49 ± 9.24	0.210^a^	—
LATEF (%)	65.46 (61.02, 69.68)	58.06 ± 5.15	57.24 ± 4.44	**< 0.001** ^b^	0 vs. 1: < 0.001; 0 vs. 2: < 0.001
LAPEF (%)	41.81 (39.63, 43.62)	38.20 ± 2.58	37.90 ± 2.20	**< 0.001** ^b^	0 vs. 1: < 0.001; 0 vs. 2: < 0.001
LAAEF (%)	40.65 (34.91, 46.22)	32.13 ± 5.43	31.29 ± 4.68	**< 0.001** ^b^	0 vs. 1: < 0.001; 0 vs. 2: < 0.001

*Note:* Statistical methods: Post hoc analysis: Bonferroni correction applied for pairwise comparisons. *p* < 0.05 was considered to indicate statistical significance, Bold values denote statistically significant differences (*p* < 0.05). Groups: 0 = control; 1 = short‐duration HIV group; 2 = HIV long‐duration HIV group.

Abbreviations: CI, cardiac index; CO, Cardiac Output; CVi, cellular volume index; ECMVi, extracellular matrix volume index; ECV, extracellular volume; EDVi, end‐diastolic volume index; EF, ejection fraction; ESVi, end‐systolic volume index; LAAEF, active left atrial ejection fraction; LAPEF, passive left atrial ejection fraction; LATEF, total left atrial ejection fraction; LAV pre‐A, left atrial pre‐atrial contraction volume; LAVmax, left atrial maximum volume; LAVmin, left atrial minimum volume; LV, left ventricular; LVM, left ventricular mass; MMi, myocardial mass index; MR, magnetic resonance; MVR, mass‐to‐volume ratio; SV, stroke volume; SVi, stroke volume index.

^a^ One‐way ANOVA, mean ± SD.

^b^ Kruskal–Wallis *H* test, median (Q1, Q3).

### 3.3 Structural and Functional Parameters of the Left Ventricle and Left Atrium

The LVEDVi was significantly lower in the long‐duration HIV group (65.44 ± 13.21 mL/m^2^) than in the control group (73.42 ± 10.99 mL/m^2^) and the short‐duration HIV group (72.08 ± 10.62 mL/m^2^). The myocardial mass‐to‐volume ratio (MVR) was significantly greater in the long‐duration HIV group (0.78 ± 0.16 g/mL) than in the control group (0.61 ± 0.11 g/mL) and the short‐duration group (median 0.63 g/mL [IQR: 0.55, 0.69]). Furthermore, the LVM was significantly greater in the long‐duration HIV group (90.13 ± 24.06 g) than in the control group (73.29 ± 17.06 g), with no significant differences detected between the control and short‐duration HIV group (*p* = 0.609) or between the short‐ and long‐duration HIV groups (*p* = 0.061). No significant differences in the LVEF, SVi and CI were detected among the groups (*p* = 0.981, *p* = 0.206, and *p* = 0.052, respectively) (Table 2).

The LAVmin was significantly greater in both the short‐duration HIV group (23.44 ± 6.63 mL) and the long‐duration HIV group (23.79 ± 6.87 mL) than in the control group (18.25 ± 6.68 m), although there was no significant difference between the long‐ and short‐duration HIV groups (*p* = 0.812). Additionally, LAVmax and LAVpre‐A did not significantly differ among the groups (*p* = 0.797 and *p* = 0.210, respectively). LATEF was significantly lower in both the long‐ and short‐duration HIV groups (short duration: 58.06% ± 5.15%; long duration: 57.24% ± 4.44%) than in the control group (median 65.46% [IQR: 61.02, 69.68]), but there was no significant difference between the long‐ and short‐duration HIV groups (*p* = 0.271). Additionally, both LAPEF and LAAEF were significantly lower in the long‐ and short‐duration HIV groups than in the control group (Table 2).

### 3.4 Myocardial Strain Parameters of the LA and LV

LV‐GRS was significantly lower in both the short‐ and long‐duration HIV groups than in the control group (median 33.16% [IQR: 28.44–37.52] and 29.82% ± 6.28% vs. 36.18% ± 6.39%). A significant difference in the LV‐GRS was also observed between the short‐ and long‐duration HIV groups. Additionally, LV‐GLS was significantly lower in the short‐duration HIV group (median 16.3% [IQR: −18.22, −14.05]) than in the control group (−17.85% ± 2.57%), as was LV‐GCS (−17.65% ± 1.98% vs. median −19.11% [IQR: −20.47, −17.66]). No significant differences in the LV‐GLS or LV‐GCS were detected between the short‐ and long‐duration HIV groups (*p* = 0.237 and *p* = 0.052, respectively). Myocardial strain rate analysis showed that the LV radial peak systolic strain rate (LV SRs‐R) and LV radial early diastolic strain rate (LV SRe‐R) were both significantly lower in the short‐ and long‐duration HIV groups than in the control group. In contrast, no significant intergroup differences were observed in circumferential strain rates, including peak systolic strain rate (*p* = 0.914) and early diastolic strain rate (*p* = 0.798) or in longitudinal strain rates, including peak systolic strain rate (*p* = 0.401) and early diastolic strain rate (*p* = 0.236). Moreover, the LA PGLSt was significantly lower in the long‐duration HIV group (median −15.10% [IQR: −18.55, −11.48]) than in the control group (median −9.4% [IQR: −18.00, 38.22]), with no significant difference between the short‐ and long‐duration HIV groups (*p* = 0.153) (Table 3).

**TABLE 3:** Myocardial strain parameters of the LA and LV in the control and HIV groups.

Variables	Control group (*N* = 34)	Short‐duration HIV group (*N* = 43)	Long‐duration HIV group (*N* = 40)	*p*	Post hoc pairwise comparison *p*
LV‐GRS (%)	36.18 ± 6.39	33.16 (28.44, 37.52)	29.82 ± 6.28	**< 0.001**	0 vs. 1: 0.025; 0 vs. 2: < 0.001; 1 vs. 2: 0.035
LV‐GCS (%)	−19.11 (−20.47, −17.66)	−17.65 ± 1.98	−18.65 ± 2.20	**0.028**	0 vs. 1: 0.011
LV‐GLS (%)	−17.85 ± 2.57	−16.30 (−18.22, −14.05)	−16.97 ± 3.15	**0.033**	0 vs. 1: 0.009
LV SRs‐R (1/s)	2.13 ± 0.49	1.78 (1.42, 2.21)	1.67 ± 0.42	**0.001**	0 vs. 1: 0.012; 0 vs. 2: < 0.001
LV SRs‐C (1/s)	−1.07 (−1.22, −1.01)	−1.15 (−1.35, −0.99)	−1.14 ± 0.45	0.914	—
LV SRs‐L (1/s)	−1.04 ± 0.17	−1.07 (−1.31, −0.83)	−0.99 (−1.17, −0.87)	0.401	—
LV SRe‐R (1/s)	−2.15 ± 0.71	−1.65 (−2.01, −1.24)	−1.56 ± 0.44	**0.001**	0 vs. 1: 0.009; 0 vs. 2: < 0.001
LV SRe‐C (1/s)	0.83 (0.67, 0.91)	0.79 (0.63, 1.13)	0.75 (0.63, 1.02)	0.798	—
LV SRe‐L (1/s)	0.91 ± 0.24	0.78 (0.62, 1.04)	0.77 (0.64, 0.93)	0.236	—
LA PGLSt (%)	−9.40 (−18.00, 38.22)	−13.50 (−16.90, −8.20)	−15.10 (−18.55, −11.48)	**0.032**	0 vs. 2: 0.009

*Note:* Statistical methods: Data are presented as medians (lower quartile, upper quartile). Group comparisons were performed using the Kruskal–Wallis test, with post hoc pairwise comparisons adjusted by Bonferroni correction. *p* < 0.05 was considered to indicate statistical significance, Bold values denote statistically significant differences (*p* < 0.05). Groups: 0 = control; 1 = short‐duration HIV group; 2 = long‐duration HIV group.

Abbreviations: C, circumferential; GCS, global circumferential strain; GLS, global longitudinal strain; GRS, global radial strain; L, longitudinal; LA PGLSt, left atrial peak global longitudinal strain; LA, left atrium; LV, left ventricle; R, radial; SRe, early diastolic strain rate; SRs, systolic strain rate.

### 3.5 Correlation Analysis

**FIGURE 2:**
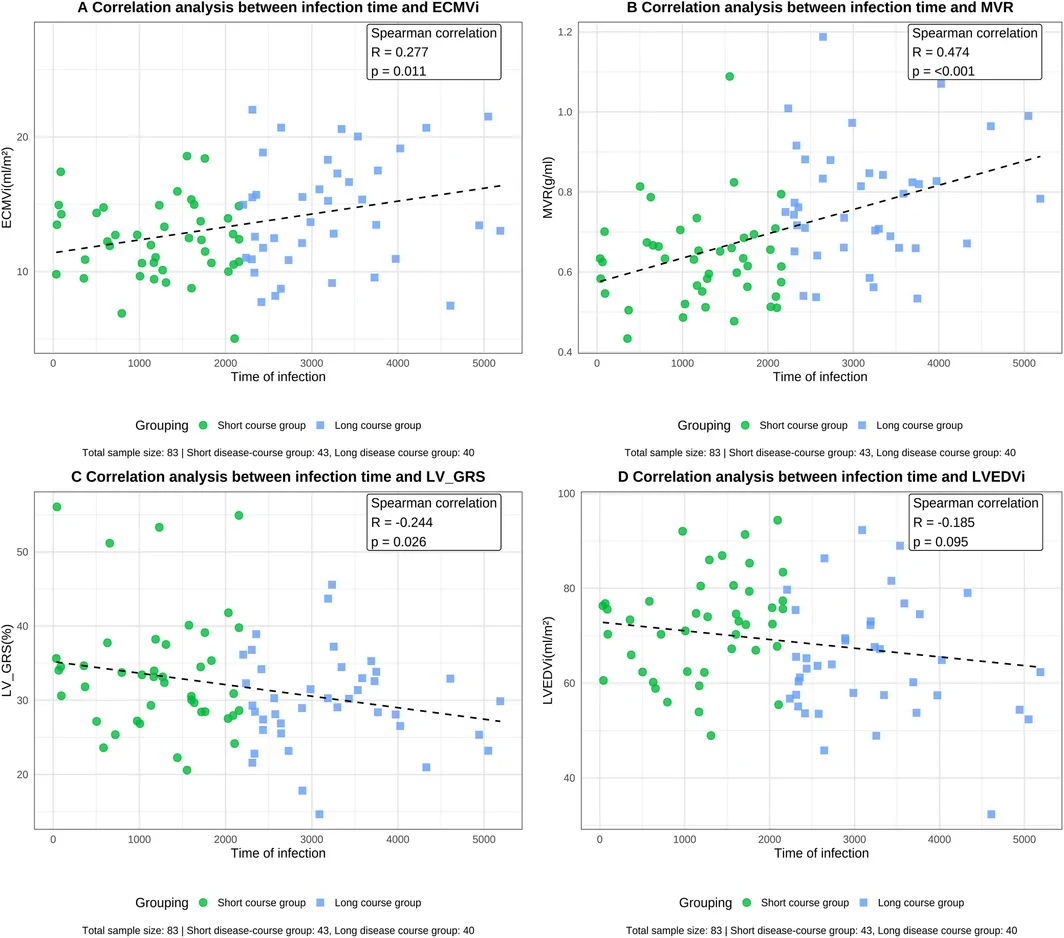
Scatter plots showing correlations between infection time: (A) the ECMVi, (B) the MVR, (C) the LV‐GRS, and (D) the LVEDVi. ECMVi, extracellular matrix volume index; LVEDVi, left ventricular end‐diastolic volume index; LV‐GRS, left ventricular global radial strain; MVR, myocardial mass‐to‐volume ratio.

The duration of HIV infection was significantly positively correlated with the ECVMi (*r* = 0.277) and MVR (*r* = 0.474) but significantly negatively correlated with the LV‐GRS (*r* = −0.244). In contrast, no significant association between the HIV infection duration and the LVEDVi was detected (*p* = 0.095) (Figure 2).

### 3.6 Logistic Regression Predicted Myocardial Injury Progression in HIV Patients

**FIGURE 3:**
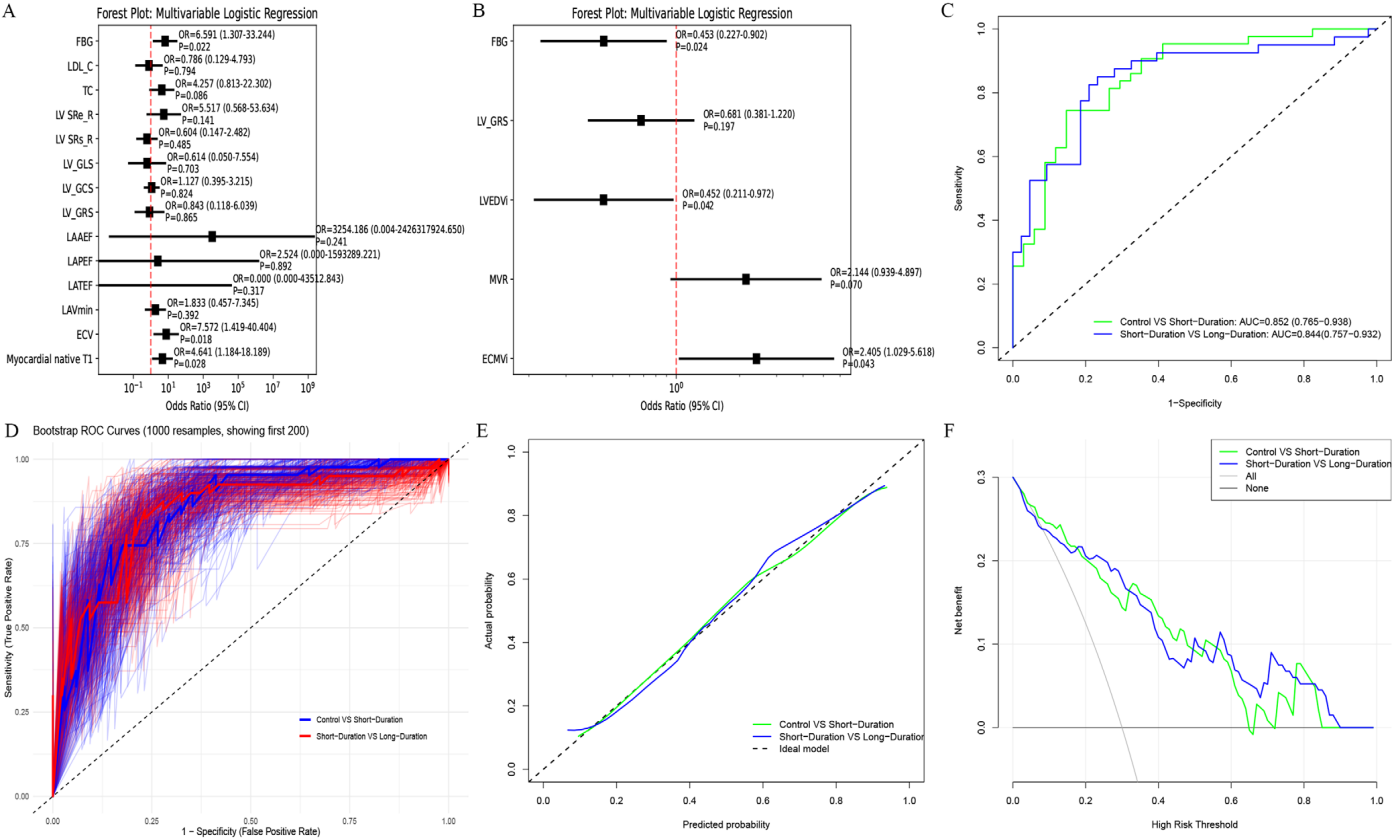
Forest plots display multivariable logistic regression results for HIV duration groups: (A) control versus short‐duration, (B) short‐ versus long‐duration. (C) ROC curves show model performance (AUCs: 0.852 for short vs. control, 0.844 for long vs. short). (D) Bootstrap curves, (E) calibration curves, and (F) decision curves are also provided. ECMVi, extracellular matrix volume index; ECV, extracellular volume; FBG, fasting blood glucose; LAAEF, active left atrial ejection fraction; LAPEF, passive left atrial ejection fraction; LATEF, total left atrial ejection fraction; LAVmin, left atrial minimum volume; LDL‐C, low‐density lipoprotein cholesterol; LV SRe‐R, left ventricular radial early diastolic strain rate; LV SRs‐R, left ventricular radial systolic strain rate; LVEDVi, left ventricular end‐diastolic volume index; LV‐GCS, left ventricular global circumferential strain; LV‐GLS, left ventricular global longitudinal strain; LV‐GRS, left ventricular global radial strain; MVR, mass‐to‐volume ratio; TC, total cholesterol.

In the multifactorial logistic regression analysis, predictors including myocardial native T1, ECV, LAVmin, LATEF, LAPEF, LAAEF, LV‐GRS, LV‐GCS, LV‐GLS, LV SRs‐R, LV SRe‐R, FBG, TC, and LDL‐C were analyzed to discriminate between the short‐duration HIV group and the control group. Three predictors were significant: ECV (OR = 7.572), myocardial native T1 (OR = 4.641) and FBG (OR = 6.591). The combined model (cutoff probability = 0.567) yielded an AUC of 0.852 (95% CI: 0.765–0.938), bootstrap mean AUC = 0.852 (95% CI: 0.754–0.930), with an accuracy of 79.2%, sensitivity of 74.4%, specificity of 85.3%, PPV of 86.5%, and NPV of 72.5%. The calibration curves showed excellent agreement, and the decision curve analysis confirmed a positive net benefit across threshold probabilities of 0.1–0.65 (Figure 3).

Next, predictors including LVEDVi, MVR, ECMVi, LV‐GRS, and FBG were analyzed to differentiate between long‐ and short‐duration HIV groups. Three predictors were significant: LVEDVi (OR = 0.452), ECMVi (OR = 2.405), and FBG (OR = 0.453). The combined model (cutoff probability = 0.443) yielded an AUC of 0.844 (95% CI: 0.757–0.932); the bootstrap mean AUC was 0.843 (95% CI: 0.755–0.923), with an accuracy of 80.7%, sensitivity of 85.0%, specificity of 76.7%, PPV of 77.3%, and NPV of 84.6%. The calibration was strong, and the decision curve analysis showed substantial net benefit across thresholds of 0.1–0.9 (Figure 3).

### 3.7 Reproducibility

**FIGURE 4:**
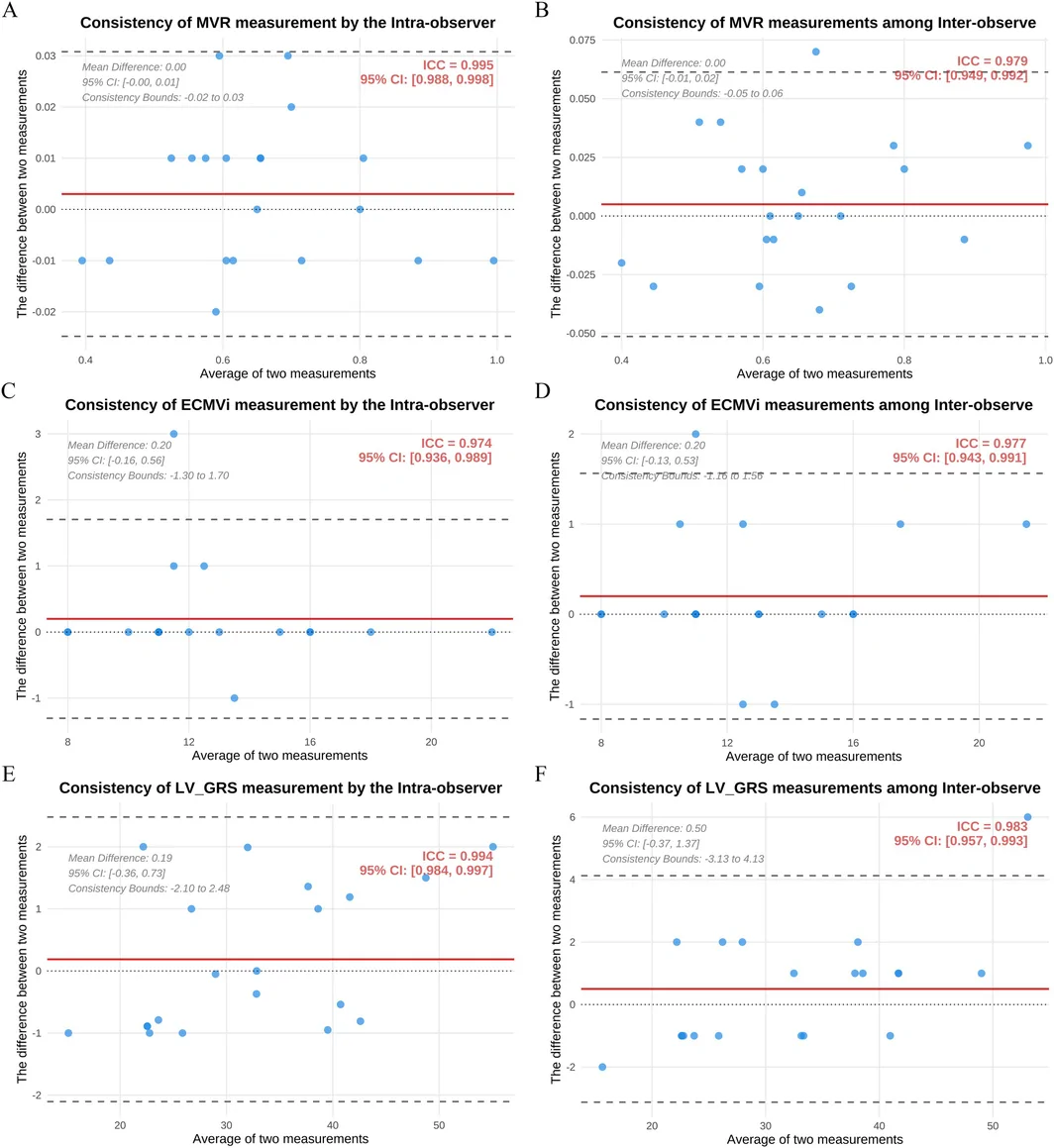
Bland–Altman analysis demonstrates good agreement in measurements of MVR (intra‐observer: A, inter‐observer: B), ECMVi (intra‐observer: C, inter‐observer: D), and LV‐GRS (intra‐observer: E, inter‐observer: F). ECMVi, extracellular matrix volume index; LV‐GRS, left ventricle global radial strain; MVR, mass‐to‐volume ratio.

Reproducibility was excellent for ECV, ECMVi, MVR, LV EDVi, LVM, LAVmin, LAPEF, LAAEF, LV‐GRS, LV SRs‐R, and LA PGLSt (ICC ≥ 0.90) and good to excellent for myocardial native T1, blood native T1, LATEF, LV‐GCS, LV‐GLS, and LV SRe‐R (ICC 0.75–0.89). LV‐GCS showed moderate reproducibility (intra‐observer ICC = 0.754; inter‐observer ICC = 0.733). Bland–Altman analysis and scatter plots for ECMVi, MVR, and LV‐GRS in Figure 4 confirm good reproducibility. Detailed ICC values with 95% confidence intervals are in Table 4.

**TABLE 4:** Inter‐observer and intra‐observer reproducibility of cardiac MRI‐derived quantitative parameters (*n* = 20).

Parameters	Intra‐observer reproducibility		Inter‐observer reproducibility	
	ICC	95% CI	ICC	95% CI
Myocardial native T1 (ms)	0.790	0.547–0.911	0.803	0.573–0.916
Blood native T1 (ms)	0.889	0.728–0.955	0.861	0.670–0.944
ECV (%)	0.982	0.955–0.993	0.979	0.946–0.992
ECMVi (mL/m^2^)	0.974	0.936–0.989	0.984	0.961–0.994
MVR (g/mL)	0.995	0.988–0.998	0.979	0.949–0.992
LVEDVi (mL/m^2^)	0.967	0.919–0.987	0.957	0.895–0.983
LVM (g)	0.993	0.984–0.997	0.970	0.926–0.988
LAVmin (mL)	0.980	0.949–0.992	0.958	0.896–0.983
LATEF (%)	0.945	0.869–0.978	0.883	0.731–0.952
LAPEF (%)	0.993	0.982–0.997	0.991	0.977–0.996
LAAEF (%)	0.958	0.898–0.983	0.916	0.803–0.966
LV‐GRS (%)	0.994	0.984–0.997	0.983	0.957–0.993
LV‐GCS (%)	0.754	0.478–0.895	0.733	0.449–0.884
LV‐GLS (%)	0.833	0.627–0.930	0.821	0.605–0.925
LV SRs‐R (1/s)	0.828	0.618–0.928	0.823	0.608–0.926
LV SRe‐R (1/s)	0.976	0.939–0.990	0.902	0.766–0.960
LA PGLSt (%)	0.961	0.904–0.984	0.944	0.867–0.977

Abbreviations: CI, confidence interval; ECMVi, extracellular matrix volume index; ECV, extracellular volume; ICC, intraclass correlation coefficient; LA PGLSt, left atrial peak global longitudinal strain during reservoir phase; LAAEF, left atrial active ejection fraction; LAPEF, left atrial passive ejection fraction; LATEF, left atrial total ejection fraction; LAVmin, left atrial minimum volume; LV SRe‐R, left ventricular radial early diastolic strain rate; LV SRs‐R, left ventricular radial systolic strain rate; LVEDVi, left ventricular end‐diastolic volume index; LV‐GCS, left ventricular global circumferential strain; LV‐GLS, left ventricular global longitudinal strain; LV‐GRS, left ventricular global radial strain; LVM, left ventricular mass; MVR, myocardial volume ratio.

## 4 Discussion

This study used cardiac MRI to show the evolution of subclinical cardiac injury in asymptomatic individuals and to identify imaging biomarkers of disease progression. Traditionally, PLWH are generally considered a homogeneous group, and the impact of the temporal dimension of disease progression on the cardiac system is often neglected. The use of a 5‐year cut‐off facilitates the distinction between short‐term and long‐term effects, thereby enabling a more precise assessment of disease progression or treatment response [15, 16]. By combining MR‐related parameters and quantitative tissue characterization, the structural and functional remodeling characteristics of the left atrioventricular system at the two duration stages were investigated and early‐stage imaging biomarkers of subclinical myocardial damage determined.

In terms of metabolic‐related indicators, compared with the control group, both the long‐duration and short‐duration HIV infection groups had significantly higher levels of TC and LDL‐C. These findings are consistent with those of previous studies, and they may be associated with lipid metabolism disorders induced by antiretroviral therapy (ART) and chronic inflammation [17]. FBG levels were significantly higher in both HIV‐infected groups than in the control group. Notably, FBG levels were higher in the short‐duration HIV group than in the long‐duration group, which may be attributed to a specific glucose metabolism stress response triggered by ART in the early stages of infection, with this effect gradually diminishing with disease progression [18, 19].

In terms of MRI myocardial tissue characteristics, compared with control individuals, all HIV‐infected individuals exhibited higher ECV and native myocardial T1 values, suggesting persistent fibrosis and chronic inflammatory responses in the myocardium of HIV patients. However, native T1 is inherently non‐specific, reflecting a composite of interstitial fibrosis, edema, amyloid deposition, and fat accumulation; consequently, elevated values alone cannot definitively indicate active inflammation [20]. In contrast, ECV serves as a more specific quantitative biomarker of interstitial expansion, predominantly reflecting collagenous fibrosis after accounting for confounding factors (e.g., edema, amyloid) [21]. The observed elevation in ECV aligns with evidence of diffuse non‐ischemic fibrosis in PLWH and likely contributes significantly to native T1 prolongation. While chronic immune activation and ART‐related mechanisms (e.g., TGF‐β1 signaling) likely promote fibrogenesis [3, 22, 23], the relative contributions of persistent low‐grade inflammation and established fibrosis to native T1 changes cannot be distinguished without complementary edema‐sensitive sequences such as T2 mapping [24]. Multivariable analysis confirms that ECV effectively differentiates PLWH from controls, underscoring its utility in risk stratification independent of disease duration. Multivariable analysis further confirmed that ECV, native T1, and FBG were independent predictors differentiating the short‐duration HIV group from controls (AUC = 0.852). These findings underscore the utility of ECV and native T1 in risk stratification independent of disease duration, while also highlighting a potential metabolic contribution from FBG. The absence of progressive ECV elevation with longer HIV duration suggests that fibrosis stems from persistent immune activation rather than the duration of infection [25]. Notably, ECMVi was significantly higher in the long‐duration HIV group and positively correlated with the duration of infection, potentially offering enhanced sensitivity for tracking absolute interstitial expansion over time.

In terms of left ventricular structural remodeling, this study showed a concentric remodeling pattern in the left ventricle of HIV‐infected individuals, characterized by a significantly lower LVEDVi in the long‐duration HIV group than in both the short‐duration HIV group and the control group. Additionally, the MVR was greater in the long‐duration HIV group than in the short‐duration group and the control group and was positively correlated with the duration of HIV infection. Although this type of remodeling pattern is common in individuals with pressure overload conditions such as hypertension or metabolic diseases [26], it has rarely been reported in HIV‐infected individuals. However, the CVi did not significantly differ among the groups, suggesting that myocardial hypertrophy does not originate from cellular proliferation but is secondary to an increase in myocardial mass due to extracellular matrix expansion, which represents non‐hemodynamically driven structural remodeling mediated by chronic inflammation [5]. Although correlation analysis did not show a direct linear relationship between LVEDVi and infection duration across all individuals, its significant decline in the long‐duration group highlights its role in staging the disease. This concentric remodeling pattern contrasts with ventricular dilation in end‐stage heart failure [27], likely due to differences in ART timing, immune status, and comorbidities [28].

A key innovation arising from the comparison between long‐ and short‐duration HIV infection is a combined model incorporating LVEDVi, ECMVi, and FBG, which achieved high diagnostic accuracy (AUC = 0.844). This contrasts with prior reports that found “no associations between HIV disease severity and CMR metrics” when severity was defined by traditional markers such as CD4 count [10]. The inverse association of FBG with longer HIV duration suggests a novel metabolic aspect in which glycemic status may shape cardiac adaptation in chronic HIV, pointing to metabolic‐inflammatory crosstalk [29]. The strong calibration and net benefit of the duration‐stratified model support its utility, addressing gaps resulting from varied fibrosis estimates.

Analysis of left ventricular myocardial strain mechanics revealed a unique, duration‐dependent pattern of functional changes. LV‐GRS was significantly lower in HIV patients, decreasing stepwise and correlating negatively with disease duration. LV‐GLS and LV‐GCS abnormalities were mainly in the short‐duration group, with some “recovery” in the long‐duration group. This pattern—early longitudinal and circumferential involvement, then progressive radial deterioration—differs from uniform strain injury in hypertension or diabetes [30]. These findings indicate HIV‐related cardiomyopathy may involve a distinct pathway, with early subendocardial impairment affecting longitudinal motion [31]. Later leading to fibrosis and reduced radial contractile force. Despite reduced LV SRs‐R and LV SRe‐R in all HIV‐infected individuals, LVEF showed no significant difference, aligning with subclinical cardiac dysfunction [32].

Analysis of the structure and function of the left atrium showed that, compared with the control group, both the long‐duration and short‐duration HIV groups exhibited increased LAVmin values and decreased LATEF. Both the active and passive ejection fractions also decreased. However, there were no significant differences in these indicators between the long‐duration and short‐duration HIV groups. These findings suggest that HIV‐infected patients experience left atrial remodeling and dysfunction, which is generally consistent with previous results [8]. This remodeling is closely related to chronic inflammation, immune dysregulation, and accelerated aging, thus representing a typical feature of HIV‐related cardiac lesions [8]. Notably, only the long‐duration HIV group had significantly abnormal LA PGLSt values, indicating that atrial myocardial deformation progressively worsened as the disease advanced. These alterations may result from increased compensatory contraction and increased myocardial stiffness [33], further confirming the role of atrial–ventricular coupling dysregulation in HIV‐related cardiac dysfunction.

## 5 Limitations

First, wide confidence intervals for key predictors, especially extracellular volume fraction (ECV), reflect limited sample size and imprecise estimates. Consequently, larger prospective studies are needed to confirm findings. Second, group differences were found, but the sample size may limit the statistical power to detect smaller effects. Third, unmeasured variables could introduce confounding bias, requiring larger studies with comprehensive data. Finally, native T1 mapping cannot distinguish fibrosis from edema, necessitating cautious interpretation and more specific techniques such as T2 mapping.

## 6 Conclusions

This study delineates the temporal evolution of left ventricular structure and function in the setting of asymptomatic HIV infection, revealing a distinct biphasic trajectory. The early stage is characterized by subclinical myocardial inflammation, manifested by elevated extracellular volume (ECV) and impaired longitudinal and circumferential strain. With disease progression, the phenotype transitions to concentric remodeling, marked by increased ECMVi, reduced LVEDVi, and a gradual deterioration in radial function. Notably, the combined model incorporating ECV, native T1, and FBG emerges as a robust predictive tool for differentiating early‐stage infection from healthy controls, while a multimodal model integrating LVEDVi, ECMVi, and FBG demonstrates promising utility for stratifying infection duration.

## Funding

This work was supported by the Medical Science and Technology Research Program of Chongqing Banan Science and Technology Bureau and Chongqing Banan Health Commission (Grant Nos. WJ202300124 and BNWJ202500153).

## References

[1] L. M. Obare , T. Temu , S. A. Mallal , and C. N. Wanjalla , “Inflammation in HIV and Its Impact on Atherosclerotic Cardiovascular Disease,” Circulation Research 134 (2024): 1515–1545, 10.1161/circresaha.124.323891. 38781301 PMC11122788

[2] I. Pinos , A. Blanco , J. Kelschenbach , et al., “EcoHIV Infection Promotes Atherosclerosis Progression in LDLR‐Deficient Mice,” Arteriosclerosis, Thrombosis, and Vascular Biology 45 (2025): e470–e482, 10.1161/atvbaha.125.323004. 40905117 PMC12700074

[3] Y. Kato , B. Ambale‐Venkatesh , M. Naveed , et al., “HIV, HIV‐Specific Factors, and Myocardial Disease in Women,” Clinical Infectious Diseases 79 (2024): 451–461, 10.1093/cid/ciae077. 38356158 PMC11327791

[4] I. Nazari and M. J. Feinstein , “Evolving Mechanisms and Presentations of Cardiovascular Disease in People With HIV: Implications for Management,” Clinical Microbiology Reviews 37 (2024): e0009822, 10.1128/cmr.00098-22. 38299802 PMC10938901

[5] E. Teer , L. Dominick , N. C. Mukonowenzou , and M. F. Essop , “HIV‐Related Myocardial Fibrosis: Inflammatory Hypothesis and Crucial Role of Immune Cells Dysregulation,” Cells 11 (2022): 11, 10.3390/cells11182825. PMC9496784 36139400

[6] P. de Leuw , C. T. Arendt , A. E. Haberl , et al., “Myocardial Fibrosis and Inflammation by CMR Predict Cardiovascular Outcome in People Living With HIV,” JACC: Cardiovascular Imaging 14 (2021): 1548–1557, 10.1016/j.jcmg.2021.01.042. 33865770

[7] W. Ding , L. Li , J. Qiu , et al., “CineMyoPS: Segmenting Myocardial Pathologies From Cine Cardiac MR,” IEEE Transactions on Medical Imaging 44 (2025): 5027–5037, 10.1109/tmi.2025.3586254. 40622836

[8] T. E. Peterson , V. S. Hahn , R. Moaddel , et al., “Proteomic Signature of HIV‐Associated Subclinical Left Atrial Remodeling and Incident Heart Failure,” Nature Communications 16 (2025): 610, 10.1038/s41467-025-55911-0. PMC11725572 39800750

[9] S. G. Shitole , J. M. Lazar , C. C. Taub , et al., “Human Immunodeficiency Virus and Cardiac End‐Organ Damage in Women: Findings From an Echocardiographic Study Across the United States,” Clinical Infectious Diseases 76 (2023): 210–219, 10.1093/cid/ciac795. 36184972 PMC10202437

[10] K. C. Wu , S. A. Haberlen , M. W. Plankey , et al., “Human Immunodeficiency Viral Infection and Differences in Interstitial Ventricular Fibrosis and Left Atrial Size,” European Heart Journal Cardiovascular Imaging 22 (2021): 888–895, 10.1093/ehjci/jeab037. 33693554 PMC8291675

[11] M. W. Han , P. H. Kim , C. H. Suh , et al., “Risk of Acute Kidney Injury Following Gadolinium‐Based Contrast Agent‐Enhanced MRI: Propensity‐Matched 7‐Year Cohort Study,” Investigative Radiology 61 (2026): 334–340, 10.1097/rli.0000000000001239. 40977576

[12] D. Du Bois and E. F. Du Bois , “A Formula to Estimate the Approximate Surface Area if Height and Weight Be Known. 1916,” Nutrition 5 (1989): 303–311, discussion 312–313. 2520314

[13] N. Kawel‐Boehm , S. J. Hetzel , B. Ambale‐Venkatesh , et al., “Society for Cardiovascular Magnetic Resonance Reference Values (“Normal Values”) in Cardiovascular Magnetic Resonance: 2025 Update,” Journal of Cardiovascular Magnetic Resonance 27 (2025): 101853, 10.1016/j.jocmr.2025.101853. 39914499 PMC12159681

[14] T. K. Koo and M. Y. Li , “A Guideline of Selecting and Reporting Intraclass Correlation Coefficients for Reliability Research,” Journal of Chiropractic Medicine 15 (2016): 155–163, 10.1016/j.jcm.2016.02.012. 27330520 PMC4913118

[15] J. Stover , R. Glaubius , Y. Teng , et al., “Modeling the Epidemiological Impact of the UNAIDS 2025 Targets to End AIDS as a Public Health Threat by 2030,” PLoS Medicine 18 (2021): e1003831, 10.1371/journal.pmed.1003831. 34662333 PMC8559943

[16] K. M. Mitchell , M. Maheu‐Giroux , D. Dimitrov , et al., “How Can Progress Toward Ending the Human Immunodeficiency Virus Epidemic in the United States be Monitored?,” Clinical Infectious Diseases 75 (2022): 163–169, 10.1093/cid/ciab976. 34849635 PMC9403299

[17] D. M. Kirkegaard‐Klitbo , M. T. Thomsen , M. Gelpi , F. Bendtsen , S. D. Nielsen , and T. Benfield , “Hepatic Steatosis Associated With Exposure to Elvitegravir and Raltegravir,” Clinical Infectious Diseases 73 (2021): e811–e814, 10.1093/cid/ciab057. 33493297

[18] J. R. Koethe , C. Moser , T. T. Brown , et al., “Adipokines, Weight Gain and Metabolic and Inflammatory Markers After Antiretroviral Therapy Initiation: AIDS Clinical Trials Group (ACTG) A5260s,” Clinical Infectious Diseases 74 (2022): 857–864, 10.1093/cid/ciab542. 34117756 PMC8906713

[19] F. Mikaeloff , S. Svensson Akusjärvi , G. M. Ikomey , et al., “Trans Cohort Metabolic Reprogramming Towards Glutaminolysis in Long‐Term Successfully Treated HIV‐Infection,” Communications Biology 5 (2022): 27, 10.1038/s42003-021-02985-3. 35017663 PMC8752762

[20] V. Nauffal , M. D. R. Klarqvist , M. C. Hill , et al., “Noninvasive Assessment of Organ‐Specific and Shared Pathways in Multi‐Organ Fibrosis Using T1 Mapping,” Nature Medicine 30 (2024): 1749–1760, 10.1038/s41591-024-03010-w. PMC12574816 38806679

[21] H. J. Lee , H. Lee , S. M. Kim , et al., “Diffuse Myocardial Fibrosis and Diastolic Function in Aortic Stenosis,” JACC: Cardiovascular Imaging 13 (2020): 2561–2572, 10.1016/j.jcmg.2020.07.007. 32828787

[22] O. Chehab , A. Abdollahi , S. P. Whelton , et al., “Association of Lipoprotein(a) Levels With Myocardial Fibrosis in the Multi‐Ethnic Study of Atherosclerosis,” Journal of the American College of Cardiology 82 (2023): 2280–2291, 10.1016/j.jacc.2023.10.016. 38057070 PMC11730445

[23] R. K. Dutta , S. Chinnapaiyan , M. J. Santiago , I. Rahman , and H. J. Unwalla , “Gene‐Specific MicroRNA Antagonism Protects Against HIV Tat and TGF‐β‐Mediated Suppression of CFTR mRNA and Function,” Biomedicine & Pharmacotherapy 142 (2021): 112090, 10.1016/j.biopha.2021.112090. 34463266 PMC9100877

[24] K. Chaikriangkrai , M. A. Abbasi , R. Sarnari , et al., “Prognostic Value of Myocardial Extracellular Volume Fraction and T2‐Mapping in Heart Transplant Patients,” JACC: Cardiovascular Imaging 13 (2020): 1521–1530, 10.1016/j.jcmg.2020.01.014. 32199848 PMC8809107

[25] C. Yan , R. Li , X. Guo , et al., “Cardiac Involvement in Human Immunodeficiency Virus Infected Patients: An Observational Cardiac Magnetic Resonance Study,” Frontiers in Cardiovascular Medicine 8 (2021): 756162, 10.3389/fcvm.2021.756162. 34869667 PMC8634394

[26] B. Alexandre‐Santos , G. D. S. Reis , G. R. Medeiros , et al., “Bisphenol S Exposure Induces Cardiac Remodeling and Aggravates High‐Fat Diet‐Induced Cardiac Hypertrophy in Mice,” Environmental Research 261 (2024): 119781, 10.1016/j.envres.2024.119781. 39142458

[27] J. A. Hudson , E. D. Majonga , R. A. Ferrand , P. Perel , S. R. Alam , and A. S. V. Shah , “Association of HIV Infection With Cardiovascular Pathology Based on Advanced Cardiovascular Imaging: A Systematic Review,” Journal of the American Medical Association 328 (2022): 951–962, 10.1001/jama.2022.15078. 36098725 PMC9471974

[28] M. Valero‐Muñoz , E. L. Saw , D. R. Pimentel , et al., “Molecular Insights in HIV‐Associated Cardiac Dysfunction,” Communications Medicine (London) 6 (2025): 87, 10.1038/s43856-025-01349-w. PMC12877094 41476135

[29] K. Maswanganyi , A. Khathi , and M. Gamede , “HIV‐Pharmacotherapy and Pathogenesis of Diabetes‐Induced Cardiovascular Complications: An Updated Narrative Review,” Cardiovascular Research 122 (2026): 446–466, 10.1093/cvr/cvag036. 41618817

[30] C. J. Pua , G. Loo , M. Kui , et al., “Impact of Diabetes on Myocardial Fibrosis in Patients With Hypertension: The REMODEL Study,” Circulation. Cardiovascular Imaging 16 (2023): 545–553, 10.1161/circimaging.123.015051. 37431660 PMC10351902

[31] S. Sivapathan , P. Geenty , T. Deshmukh , et al., “Alterations in Multi‐Layer Strain in AL Amyloidosis,” Amyloid 29 (2022): 128–136, 10.1080/13506129.2022.2026914. 35188014

[32] A. C. Egbe , W. R. Miranda , J. A. Dearani , and H. M. Connolly , “Hemodynamics and Clinical Implications of Occult Left Ventricular Dysfunction in Adults Undergoing Ebstein Anomaly Repair,” Circulation. Cardiovascular Imaging 14 (2021): e011739, 10.1161/circimaging.120.011739. 33583197 PMC7888701

[33] M. Lisi , G. E. Mandoli , M. Cameli , et al., “Left Atrial Strain by Speckle Tracking Predicts Atrial Fibrosis in Patients Undergoing Heart Transplantation,” European Heart Journal Cardiovascular Imaging 23 (2022): 829–835, 10.1093/ehjci/jeab106. 34118154

